# Insomnıa ın Heart Faılure Patıents ın a Hospıtal Settıng: Causes, Consequences, and Interventıons

**DOI:** 10.1007/s11886-025-02256-1

**Published:** 2025-07-04

**Authors:** Mehmet Emin Atay, Bahar Çiftçi

**Affiliations:** 1 Ağrı İbrahim Çeçen University, Ağrı, Türkiye; 2 Atatürk University, Erzurum, Türkiye

**Keywords:** Heart failure, Insomnia, Hospital environment, Sleep management, Quality of life

## Abstract

**Purpose of Review:**

This review explores the causes, consequences, and management approaches of hospital-acquired insomnia in patients with heart failure (HF). It examines the key factors contributing to insomnia in hospitalized HF patients, its impact on health outcomes, and effective management strategies to address the issue.

**Recent Findings:**

Recent research highlights that environmental factors (such as noise, lighting, and medical interventions) and clinical symptoms (including dyspnea and nocturia) significantly contribute to insomnia in hospitalized HF patients. Insomnia exacerbates HF symptoms, increasing hospitalization rates, healthcare costs, and the risk of cardiovascular complications. Non-pharmacological interventions have improved sleep quality and overall well-being, including Cognitive Behavioral Therapy for Insomnia (CBT-I), sleep hygiene education, and supervised exercise programs.

**Summary:**

Hospital-acquired insomnia negatively affects both the physical and psychosocial health of HF patients. Effective management requires a multidisciplinary approach, incorporating both pharmacological and non-pharmacological strategies. Nurses play a crucial role in implementing sleep-promoting interventions. Creating a hospital environment that supports sleep, raising healthcare professionals’ awareness, and integrating evidence-based interventions can enhance recovery outcomes. Future research should focus on long-term studies evaluating the efficacy of insomnia management strategies in HF patients.

## Introductıon

Heart failure (HF) is a chronic disease in which the heart loses its capacity to pump enough blood around the body. This leads to shortness of breath, fatigue, and edema due to insufficient oxygen and nutrient transport to organs and tissues. In patients with heart failure, the load on the circulatory system increases with the weakening of the heart muscle, which causes fluid accumulation in the body. It seriously reduces patients'quality of life [1]. In such patients, sleep patterns are an essential factor affecting the course of the disease; patients are adversely affected both physically and mentally by the deterioration of sleep duration and quality. Especially the time spent in the hospital environment, additional stress factors brought on by the disease, and hospital-induced sleep disorders can negatively affect the recovery process of HF patients [2].

Sleep disorders, which are frequently observed among patients with heart failure, are an essential problem that directly affects general health. Insomnia is characterized by symptoms such as difficulty falling asleep, frequent awakenings, or early morning awakenings, and it leads to fatigue, loss of concentration, and mood changes during the day [3]. This is directly linked to patients'exposure to stress and disease burden [4]. The interplay between insomnia and heart failure may contribute to disease progression by creating additional pressure on the cardiovascular system [4]. In this process, increased insomnia symptoms reduce the quality of life of patients, as well as increase the frequency of hospitalization and healthcare costs [5].

Hospital-acquired insomnia is a significant problem, especially for individuals with severe chronic diseases such as heart failure. Insomnia in the hospital environment is caused by noise, light, constant medical interventions, and other environmental factors that disrupt sleep patterns. These factors may affect patients'ability to fall asleep and sleep, causing fatigue, stress, and mood disorders during the day [6]. For patients with heart failure, poor sleep quality makes symptom management difficult and negatively affects the recovery process. This condition also triggers psychological problems such as depression, anxiety, and increased, fatigue [3].

Insomnia has a multidimensional impact on the health of heart failure patients. These patients wake up frequently at night and do not feel sufficiently rested in the morning. This leads to poor performance in daily activities [5]. Disruptions in sleep patterns can increase the pressure on the cardiovascular system, leading to disease progression and frequent hospitalizations (Laugshospitalizations) [7]. Therefore, hospital-acquired insomnia and its broader effects are considered an essential target in the essentialist process of heart failure patients, and, managing this problem is of great importance. This study examines the causes and effects of insomnia in patients with heart failure in the hospital setting and its consequences on the general health status of patients. Hospital-acquired insomnia is seen as a problem that affects not only sleep quality but also the recovery process, cardiovascular stability, and psychosocial status of patients [3].

This study will evaluate the effects of environmental factors (light, noise, and interventions), heart failure symptoms (breathlessness, orthopnoea), and treatment modalities on sleep quality in hospital-acquired insomnia. In addition, this study will investigate the effects of insomnia on clinical outcomes, risk of hospital readmission, and quality of life. It will address the effectiveness of pharmacological and non-pharmacological sleep regulation interventions. The study's results are expected to contribute to developing evidence-based recommendations for managing hospital-acquired insomnia and optimizing the recovery process in heart failure patients.

## Causes of Insomnia in the Hospital Environment

### Physical Environment Factors

One of the most important physical factors affecting sleep patterns in the hospital environment is noise. Continuously operating medical devices, alarms of monitors, movements of other patients, and nocturnal interventions of the care team can negatively affect patients'falling asleep and sleep duration. Research shows that these environmental factors are a severe source of insomnia in patients. The increase in noise levels, especially at night, disrupts sleep continuity and makes it difficult for patients to rest, negatively affecting sleep quality [2]. In addition, continuous illumination in the hospital environment may prevent the release of melatonin hormone that supports the transition to sleep by providing night darkness. Constant exposure to artificial light disrupts the body's biological clock, making it challenging to regulate the sleep–wake cycle [3].

### Clinical Interventions and Medical Devices

Medical devices and continuous clinical interventions used in heart failure patients are other important causes of hospital-acquired insomnia. For example, interventions such as monitor alarms used for regularly monitoring patients, blood pressure measurements, and medication administration cause frequent interruptions during the night. Such interventions severely disrupt patients'sleep continuity and lead to a feeling of restlessness throughout the night [5]. Medical equipment such as oxygen devices and ventilators used especially in heart failure patients can affect both the patient's physical comfort and sleep quality. The physical discomfort and noise created by medical devices make it difficult for patients to fall asleep, causing insomnia to increase further [6].

### Impact of Disease Symptoms

Another essential factor causing insomnia in heart failure patients is the specific symptoms of the disease. For example, shortness of breath (orthopnoea) and frequent urination during the night (nocturia) disrupt the continuity of sleep. In addition to orthopnoea, paroxysmal nocturnal dyspnea (PND) is one of the most distressing and sleep-disrupting symptoms experienced by patients with heart failure. PND is characterized by sudden episodes of severe breathlessness that awaken the patient from sleep, often occurring within one to two hours after falling asleep [8]. This phenomenon is caused by fluid shifts from the periphery to the central circulation during recumbency, leading to increased pulmonary capillary pressure and interstitial edema, resulting in a sensation of suffocation or panic [9]. These awakenings are frequently accompanied by sympathetic nervous system activation, tachycardia, and heightened anxiety, which prolong wakefulness and delay sleep reinitiation [10, 11].

Recurrent PND episodes lead to sleep fragmentation, decreased total sleep time, and increased sleep latency, all of which contribute to the development or exacerbation of chronic insomnia [12]. These disturbances are strongly associated with daytime fatigue, cognitive impairment, mood instability, and reduced quality of life in heart failure patients [11]. Moreover, the anticipatory fear of breathlessness during sleep may result in maladaptive sleep behaviors such as delayed sleep initiation, sleeping in a seated position, or repeated awakenings to check breathing.

From a nursing perspective, it is crucial to recognize and assess PND as a key contributor to sleep disturbances in hospitalized patients. Sleep assessments should include questions about nighttime breathlessness, frequency of awakenings, and related anxiety. Moreover, the resolution of paroxysmal nocturnal dyspnea (PND) is strongly linked to the successful management of heart failure. Optimization of pharmacological therapy—particularly with diuretics, ACE inhibitors, and beta-blockers—can reduce pulmonary congestion, the main contributor to PND. As congestion decreases, patients often experience fewer nocturnal awakenings and improved sleep continuity, which alleviates insomnia symptoms [9, 11]. Interventions such as optimizing diuretic timing [13], managing fluid balance, and elevating the head of the bed can help reduce the burden of PND.

Moreover, effective medical management of heart failure plays a vital role in alleviating PND symptoms and improving sleep [14]. Treatments targeting the underlying hemodynamic abnormalities—such as diuretics to reduce volume overload, ACE inhibitors to improve cardiac output, and beta-blockers to lower sympathetic activity—can significantly reduce pulmonary congestion, which is the physiological basis of PND [15]. As pulmonary capillary pressure decreases and interstitial edema resolves, patients experience fewer episodes of nocturnal dyspnea. This leads to improved sleep continuity, reduced sleep anxiety, and fewer nighttime awakenings. Studies have demonstrated that as heart failure stabilizes, PND frequency and intensity decrease, resulting in better subjective sleep quality and improved daytime function [11]. Thus, heart failure treatment not only supports cardiovascular stability but also alleviates symptom-driven sleep fragmentation, forming a dual therapeutic benefit [16]. Furthermore, while dyspnea interferes with sleep initiation, nocturia contributes to frequent awakenings and poorer sleep continuity [17].

One of the primary contributors to nocturia in heart failure patients is the use of loop diuretics, which are widely prescribed to relieve fluid overload and pulmonary congestion. However, when administered in the late afternoon or evening, these medications can increase nocturnal urine production, resulting in frequent awakenings, sleep fragmentation, and difficulty resuming sleep [18]. This disruption exacerbates insomnia symptoms and decreases overall sleep efficiency, particularly among older adults and those with multiple comorbidities [6, 11].

The PLANET study and other systematic reviews emphasize that diuretics are among the most frequent pharmacological causes of nocturia, especially in patients with cardiovascular or renal diseases [18, 19]. Nocturia, in turn, is independently associated with increased fatigue, mood disturbances, falls, and reduced quality of life in heart failure patients [20].

To reduce the negative impact of diuretics on sleep, clinically supported strategies include administering them earlier in the day—preferably before 4:00 p.m.—to limit overnight diuresis, reducing fluid intake in the evening, evaluating sodium intake, and reviewing other medications that may contribute to polyuria or nocturia [9, 12]. In addition, patient education and individualized fluid/dietary plans form an essential part of nursing interventions for nocturia-associated insomnia. These integrated approaches contribute to improved sleep quality without compromising fluid management in heart failure care.

A primary contributor to nocturia in hospitalized patients with heart failure is the use of loop diuretics, which are integral for fluid overload management. However, when administered in the evening or at night, these medications increase urinary frequency during sleep hours and cause multiple awakenings. This leads to sleep fragmentation, difficulty falling back asleep, and aggravation of insomnia symptoms. Patients may also develop anticipatory anxiety about waking to void, further impairing sleep quality [21]. This pharmacologic side effect creates a paradox: while diuretics alleviate pulmonary congestion and reduce PND, they simultaneously increase nocturia, introducing a new pathway for sleep disruption. Thus, it is crucial for clinicians and nurses to evaluate the timing and dosage of diuretics to balance these competing effects [22]. These symptoms collectively prevent patients from achieving restorative sleep and may impair the recovery process. To mitigate the adverse impact of diuretic-induced nocturia on sleep, several strategies can be implemented in hospital settings. Scheduling diuretics earlier in the day—preferably before 4 p.m.—can significantly reduce nighttime urination and its disruptive effects on sleep. In addition, nurses can educate patients to limit fluid intake in the two to three hours before bedtime, unless contraindicated [6]. Regular monitoring of fluid status and individualized fluid restriction plans can also help maintain the delicate balance between congestion relief and sleep preservation. These strategies are not only practical and cost-effective but also align with person-centered nursing care. When integrated into routine clinical workflows, such interventions can substantially enhance sleep quality and support recovery in hospitalized patients with heart failure [12]. Over time, persistent nocturnal symptoms not only worsen insomnia but also undermine overall quality of life in heart failure patients [1].

In sum, when combined with environmental and physiological stressors of hospitalization, symptom-induced sleep disturbances—especially those linked to PND—can significantly impact the health and well-being of individuals living with chronic conditions like heart failure.

## Symptoms of Insomnia in Heart Failure Patients

### Common Insomnia Symptoms

Insomnia is a common problem in heart failure patients and is often manifested by symptoms such as difficulty falling asleep, difficulty maintaining sleep, and early awakening. These patients experience symptoms such as frequent awakenings during the night, sleep interruptions, and feeling tired in the morning [3]. Insomnia symptoms negatively affect not only the quality of sleep at night, but also daytime symptoms such as fatigue, loss of concentration, mood changes, and depression. A significant proportion of heart failure patients with insomnia experience fatigue and loss of energy during the day, which negatively affects quality of life by decreasing overall functionality [2].

These insomnia symptoms also reduce physical endurance in heart failure patients and make the symptoms of the disease more severe. Symptoms such as shortness of breath, chest pain, and fatigue also increase the severity of insomnia, which complicates the overall recovery process of patients [7].

### Association of Insomnia with Heart Failure Symptoms

Insomnia in heart failure patients is strongly associated with disease-specific symptoms. In particular, symptoms such as shortness of breath (orthopnoea) and frequent urination during the night (nocturia) disrupt sleep patterns, making it difficult for patients to fall asleep and stay asleep [17]. Shortness of breath increases significantly in the supine position and causes patients to change position to fall asleep constantly. This situation disrupts sleep continuity and creates constant discomfort throughout the night [23].

There is a strong interaction between heart failure symptoms and insomnia. Insomnia increases the burden on the cardiovascular system in patients with heart failure, increasing the severity of symptoms and contributing to disease progression. Studies show that heart failure patients with insomnia exhibit more depression, anxiety, and poor physical performance [12]. This situation both affects patients'general health and negatively affects the treatment processes.

## Clinical Consequences of Insomnia and its Effects on Heart Failure

### The Effects of Insomnia on Depression, Fatigue, and Quality of Life

Insomnia in patients with heart failure directly affects psychosocial outcomes such as depression and fatigue. Insomnia leads to a feeling of exhaustion and burnout during the day in these patients and also increases the severity of depressive symptoms [3]. Studies show that insomnia in heart failure patients negatively affects quality of life and that these patients experience more fatigue, anxiety, and depression [2]. In particular, depression and fatigue reduce the overall functionality of patients by reducing their participation in activities of daily living, and this leads to a significant decrease in the quality of life of patients [6]. These adverse effects caused by insomnia are essential factors that should be considered in treatment processes.

Insomnia-related fatigue may limit patients'activities of daily living by affecting cardiovascular function and physical endurance in heart failure patients. This condition affects not only physical health but also psychological health, increasing the risk of depression and anxiety [24]. Therefore, managing the effects of insomnia on depression, fatigue, and quality of life is critical in improving the overall health of heart failure patients.

### Effects on Frequency of Hospitalisation and Emergency Department Admissions

Insomnia appears to be a factor that increases the frequency of hospitalization and emergency department visits in patients with heart failure. Studies show that heart failure patients with insomnia symptoms are admitted to hospitals more frequently [12]. In particular, patients who cannot sleep at night have higher stress levels in their cardiovascular system, which leads to worsening of heart failure symptoms. As a result, failure to manage sleep disorders increases the need for hospitalization of patients, placing an additional burden on the healthcare system [25].

Increased insomnia causes heart failure patients to be admitted to emergency departments more frequently, which increases the overall health costs of patients [8]. Various studies have shown that emergency department visits are reduced in patients with insomnia management. Therefore, sleep therapy effectively reduces hospitalization rates and overall health costs [11].

### Cardiovascular Risks and Other Complications

Insomnia in heart failure patients leads to an increase in cardiovascular risk factors. Insomnia may increase the risk of cardiac events in patients with heart failure, leading to a rise in mortality rates. In a study, it was found that insomnia doubled the risk of cardiac events in heart failure patients [26]. Especially the continuation of insomnia increases the risk of hypertension by increasing blood pressure in heart failure patients and predisposes them to cardiac rhythm disorders.

In addition, it has been reported that insomnia symptoms weaken the immune system in the long term and may lead to other complications by increasing inflammatory responses [27]. These complications triggered by insomnia complicate the treatment processes of heart failure patients, worsening their general health status and further reducing their quality of life. Therefore, insomnia management is critical in maintaining cardiovascular health.

## Management of Insomnia and Interventions in Heart Failure Patients

### Pharmacological Interventions and Effects of Hypnotic Drugs

Benzodiazepines and other hypnotic drugs are commonly used to manage insomnia in heart failure patients. These medications provide temporary relief by increasing the duration of sleep, but in the long term, they create side effects such as risk of addiction, cognitive impairments, and daytime fatigue. Studies show that hypnotic drugs can alleviate short-term insomnia symptoms in patients with heart failure, but their long-term use is not recommended due to side effects [28]. Especially in patients with comorbid diseases such as heart failure, serious complications such as delirium may occur if hypnotics are withdrawn, and this may complicate sleep management in the hospital setting.

### Non-pharmacological Interventions

Non-pharmacological interventions offer effective and safe options for improving long-term sleep quality in heart failure patients. In particular, Cognitive Behavioural Therapy (CBT-I) is a frequently preferred effective treatment modality for the management of insomnia. CBT-I includes sleep hygiene improvement, stimulus control, and cognitive restructuring techniques, and studies show that this method both improves sleep quality and reduces insomnia symptoms [29]. CBT-I is also reported to be effective in reducing depression, anxiety, and fatigue in heart failure patients and contributes positively to the quality of life of these patients [30]. In addition, training on sleep hygiene includes practical solutions such as organizing patients'bedtimes, improving the sleep environment, and performing relaxation activities before sleep.

### Effects of Exercise and Rehabilitation Programmes on Sleep

Regular exercise and rehabilitation programs for patients with heart failure are another practical method to improve both sleep quality and physical health. Studies have shown that exercise has a beneficial effect on sleep. In one study, a 12-week supervised exercise program for heart failure patients improved sleep quality and physical endurance [25]. Exercise accelerates the transition to sleep, contributes to the deepening of sleep, and helps to reduce stress. In addition, it has been observed that as patients'exercise capacity increases within the scope of rehabilitation programs, the improvement in sleep quality becomes more pronounced. These various interventions make insomnia management more effective in heart failure patients. CBT-I, sleep hygiene education, and regular exercise are essential in improving these patients'physical and mental health.

## Cognitive Behavioural Therapy (CBT-I) and Other Treatment Methods

### Effectiveness of CBT-I and Ways of Implementation

Cognitive Behavioural Therapy (CBT-I) is seen as an effective treatment method to improve sleep quality in heart failure patients. CBT-I focuses on changing patients'negative thoughts about sleep and restructuring their sleep behaviors. Applications include techniques such as sleep time restriction, stimulus control, and cognitive restructuring. Research shows that CBT-I increases sleep duration and reduces insomnia severity. These effects of CBT-I improve quality of life by reducing depression, anxiety, and fatigue in patients with heart failure [31]. In addition, during CBT-I applications, patients are guided to adopt behaviors that can further improve sleep quality by receiving training on sleep hygiene [32].

Different platforms have been developed for CBT-I applications, which can be applied as group therapies or individual applications for heart failure patients. Studies have reported that CBT-I is effective in the long term for patients with heart failure and provides permanent improvements in sleep patterns [33].

### Nurses'Perceptions of CBT-I Practices and Patient Support Role

Nurses'role in treating insomnia in heart failure patients is significant. Although most nurses believe in the effectiveness of CBT-I, they state that they may encounter some difficulties in implementing this method. Especially nurses who are highly aware of the importance of sleep problems of heart failure patients support the applicability of this treatment method in the hospital environment and feel responsible for supporting patient sleep health [34]. However, it is also stated that there are some deficiencies in the training and support required to provide this practice among nurses. The ability of nurses to access CBT-I training and to offer this treatment method to patients has a significant potential to improve sleep quality.

Nurses stated that they could offer this therapy more effectively by meeting their training and support needs while applying CBT-I. The need for educational resources and guidance for nurses to provide this treatment for patients with sleep disorders was emphasized [34].

#### Other Behavioural Interventions

Apart from CBT-I, other behavioral interventions may also be effective in alleviating insomnia in patients with heart failure. Strategies such as sleep hygiene education, establishing regular bedtimes, and practicing relaxation techniques before bedtime can alleviate insomnia symptoms. These behavioral approaches enable patients to restructure and adopt sleep hygiene behaviors. In addition, short-term and low-intensity exercises have been reported to contribute to sleep quality [35].

These various behavioral interventions both improve sleep quality and reduce psychosocial symptoms such as depression, anxiety, and fatigue in patients with heart failure. Nurses can provide significant support in managing insomnia problems by being with patients in such practices.

## Future Research Directions and Clinical Applications

### Gaps in Research and New Research Areas

Studies on the effects of hospital-acquired insomnia on heart failure patients need long-term and large-scale research. For example, the long-term impact of Cognitive Behavioural Therapy (CBT-I) in patients with heart failure has not yet been thoroughly investigated. Further studies should be conducted to examine the effects of CBT-I on sleep quality, quality of life, and psychological symptoms such as depression in more depth [30]. Furthermore, studies evaluating the effectiveness of different modifications of CBT-I (e.g., group therapy or access via digital platform) on patients with heart failure are needed [30].

In addition, studies to understand the biological mechanisms of hospital-acquired insomnia are limited. In particular, investigating the effects of insomnia on the hypothalamic–pituitary–adrenal (HPA) axis and autonomic nervous system may provide valuable insights into how insomnia can be more effectively managed in these patients [36]. Studies focusing on such biological processes may help better understand the interactions between insomnia and heart failure and contribute to developing treatment strategies.

### Recommendations for Improving Insomnia Management in the Hospital Setting

Training nurses in the hospital environment about insomnia treatment is essential to improve insomnia management in heart failure patients. Increasing nurses'awareness of CBT-I applications and expanding sleep education and sleep hygiene practices for patients can enhance the quality of sleep [34]. In addition, creating sleep-friendly environments, such as reducing noise from medical devices used in the hospital environment, darkening the sleeping environment, and optimizing the sleeping arrangement, can improve the sleep quality of patients. Such arrangements may offer low-cost and practical solutions to enhance sleep quality [25].

Furthermore, observing patients'sleep patterns with digital sleep monitoring systems can help with the early diagnosis of sleep problems and optimize the timing of interventions. Such digital applications can provide a more holistic approach to insomnia management, enabling more effective monitoring of patients'sleep quality [6]. Research and applications in line with these recommendations will provide essential steps towards improving insomnia management and improving the quality of life of heart failure patients in the hospital setting [37].

## Conclusion and Recommendations

Hospital-induced insomnia is an important factor that negatively affects both the physical and psychosocial health of patients with heart failure by disrupting sleep patterns. Noise, lighting, continuous medical interventions, and specific symptoms of the disease (such as shortness of breath and frequent urination) in the hospital environment are among the main factors that trigger insomnia in these patients. Decreased sleep quality leads to problems such as depression, fatigue, and reduced quality of life, which increases the frequency of hospitalization and increases the risk of cardiovascular complications.

A multidisciplinary approach is recommended to manage hospital-acquired insomnia in heart failure patients and to support the treatment process. In this context, non-pharmacological interventions such as Cognitive Behavioural Therapy (CBT-I), sleep hygiene training, and supervised exercise programs are as important as pharmacological treatments. In addition to the supportive role nurses play in sleep management, physical arrangements necessary to create a sleep-friendly hospital environment and digital sleep monitoring systems will effectively improve sleep quality.

In this context, recommendations can be summarised as providing a sleep environment that supports patients’ recovery, increasing nurses'awareness of CBT-I and sleep management, and providing more education and resources for insomnia management in the hospital. These approaches will reduce hospital-induced insomnia in patients with heart failure, improve their general health status, and increase their quality of life.

## Key References

- Lahoz R, Fagan A, McSharry M, Proudfoot C, Corda S, Studer R. Recurrent heart failure hospitalizations increase the risk of mortality in heart failure patients with atrial fibrillation and type 2 diabetes mellitus in the United Kingdom: a retrospective analysis of Clinical Practice Research Datalink database. BMC Cardiovasc Disord. 2022;22(1):234.
  - This study underscores the rising frequency of hospitalizations among heart failure patients, emphasizing the importance of managing contributing factors such as insomnia to improve patient outcomes and reduce healthcare burdens.
- Redeker NS, Yaggi HK, Jacoby D, Hollenbeak CS, Breazeale S, Conley S, Jeon S. Cognitive behavioral therapy for insomnia has sustained effects on insomnia, fatigue, and function among people with chronic heart failure and insomnia: the HeartSleep Study. Sleep. 2022;45(1):zsab252.
  - This research provides evidence on the long-term benefits of Cognitive Behavioral Therapy for Insomnia (CBT-I) in heart failure patients, demonstrating its effectiveness in reducing fatigue and improving sleep quality, which are crucial for disease management.
- Jeon S, Conley S, Hollenbeck C, O'Connell M, Wang Z, Tocchi C, Redeker NS. Rest-activity rhythms predict the time to hospitalizations and emergency department visits among participants in a randomized controlled trial of adults with heart failure and insomnia. Sleep Med*.* 2023;108:1–7.
  - This study highlights the impact of disrupted sleep patterns on heart failure patients'hospital readmissions and emergency visits, reinforcing the need for targeted interventions to improve sleep and overall health outcomes.

## Data Availability

Availability of data and materials Datasets used and/or analyzed during the current study are available from the corresponding author upon reasonable request.
